# EGCG Inhibits Adipose-Derived Mesenchymal Stem Cells Differentiation into Adipocytes and Prevents a STAT3-Mediated Paracrine Oncogenic Control of Triple-Negative Breast Cancer Cell Invasive Phenotype

**DOI:** 10.3390/molecules26061506

**Published:** 2021-03-10

**Authors:** Narjara Gonzalez Suarez, Sahily Rodriguez Torres, Amira Ouanouki, Layal El Cheikh-Hussein, Borhane Annabi

**Affiliations:** Laboratoire d’Oncologie Moléculaire, Département de Chimie, Centre de Recherche CERMO-FC, Université du Québec à Montréal, Montréal, QC H3C 3P8, Canada; gonzalez_suarez.narjara@courrier.uqam.ca (N.G.S.); rodriguez_torres.sahily@courrier.uqam.ca (S.R.T.); ouanouki.amira@uqam.ca (A.O.); el_cheikh-hussein.layal@courrier.uqam.ca (L.E.C.-H.)

**Keywords:** adipogenesis, EGCG, triple-negative breast cancer, green tea, STAT3

## Abstract

Obese subjects have an increased risk of developing triple-negative breast cancer (TNBC), in part associated with the chronic low-grade inflammation state. On the other hand, epidemiological data indicates that increased consumption of polyphenol-rich fruits and vegetables plays a key role in reducing incidence of some cancer types. Here, we tested whether green tea-derived epigallocatechin-3-gallate (EGCG) could alter adipose-derived mesenchymal stem cell differentiation into adipocytes, and how this impacts the secretome profile and paracrine regulation of the TNBC invasive phenotype. Here, cell differentiation was performed and conditioned media (CM) from preadipocytes and mature adipocytes harvested. Human TNBC-derived MDA-MB-231 real-time cell migration was performed using the exCELLigence system. Differential gene arrays and RT-qPCR were used to assess gene expression levels. Western blotting was used to assess protein expression and phosphorylation status levels. In vitro vasculogenic mimicry (VM) was assessed with Matrigel. EGCG was found to inhibit the induction of key adipogenic biomarkers, including lipoprotein lipase, adiponectin, leptin, fatty acid synthase, and fatty acid binding protein 4. Increased TNBC-derived MDA-MB-231 cell chemotaxis and vasculogenic mimicry were observed in response to mature adipocytes secretome, and this was correlated with increased STAT3 phosphorylation status. This invasive phenotype was prevented by EGCG, the JAK/STAT inhibitors Tofacitinib and AG490, as well as upon STAT3 gene silencing. In conclusion, dietary catechin-mediated interventions could, in part through the inhibition of adipogenesis and modulation of adipocytes secretome profile, prevent the onset of an obesogenic environment that favors TNBC development.

## 1. Introduction

Adipogenesis, as defined by the formation of adipocytes from stem cells [1], and the adipose tissue itself have drawn much attention at the onset of chronic inflammation in metabolic diseases, such as type 2 diabetes mellitus, cardiovascular diseases, and in several types of cancer [2,3]. Among the processes involved in the setting of these diseases, the adipose tissue secretome has been inferred to play a crucial paracrine regulatory role in brain cancer [4], prostate cancer [5], bladder cancer [6], breast cancer [7], and colon cancer [8]. Molecular links between central obesity and breast cancer have also been inferred to trigger oncogenic signaling pathways, including NFκB, JAK, STAT3, and AKT [9].

The adipose tissue functions as an endocrine organ that, in obese people, produces a high level of tumor-promoting hormones such as leptin and estrogen, and a low level of the tumor suppressor hormone, adiponectin [10]. In particular, adipose tissues from tumor-bearing breasts have shown a distinct molecular signature and physiological status from those of tumor-free breasts [11]. Furthermore, several adipose tissue-derived miRNAs were associated with adipocyte differentiation and identified with essential roles in obesity-associated inflammation, insulin resistance, and tumor microenvironment [2]. While efficacy of currently approved drug therapies and understanding of drug mechanisms against obesity remain open for debate [12], diet-derived phytochemicals have emerged as potential candidates to combat obesity via adipose non-shivering thermogenesis [13], or targeting of the adipose tissue inflammation [14,15].

Epidemiological data indicate that increased consumption of fruits and vegetables plays a key role in reducing the incidence of some cancers [16]. These foods contain a significant number of polyphenols, which are potential agents that reduce obesity in part through reducing the amount of adipose tissue by stimulating lipolysis [17]. It has been reported that in pre-adipocyte murine models of differentiation, epigallocatechin-3-gallate (EGCG), which is the main compound in green tea, reduced adipocytes proliferation, lipid accumulation and expression of peroxisome proliferator-activated receptor gamma (PPARγ) and CCAAT/enhancer-binding protein alpha (C/EBPα) in mature adipocytes [18]. Whether diet-mediated polyphenols can consequently alter the secretome profile of adipocytes, and how secretome-mediated paracrine regulation of cancer cells’ invasive phenotype occurs, remains to be better addressed.

Therefore, the present study aims at exploring the molecular mechanisms involved in the adipocyte paracrine regulation of cancer cells invasive characteristics, and how efficient diet-derived intervention may prevent such regulation. It was found that the secretome of differentiated adipocytes specifically triggered migration in several TNBC-derived cells, but not that migration of the ovarian or colon cancer cells tested. Chronic exposure of adipose-derived mesenchymal stem cells (ADMSC) to EGCG during their differentiation into mature adipocytes effectively altered adipogenesis and the secretome profile of differentiated adipocytes as TNBC-derived cell chemotaxis and vasculogenic mimicry were inhibited. Finally, adipocyte secretome-mediated paracrine regulation of TNBC-derived cells invasive phenotype required STAT3 oncogenic signaling, and EGCG was able to acutely inhibit STAT3 phosphorylation.

## 2. Results

### 2.1. Phenotypical and Transcriptional Assessment of Adipose-Derived Mesenchymal Stem Cells Differentiation and Inhibition of Adipogenesis by Green Tea-Derived EGCG

**Figure 1:**
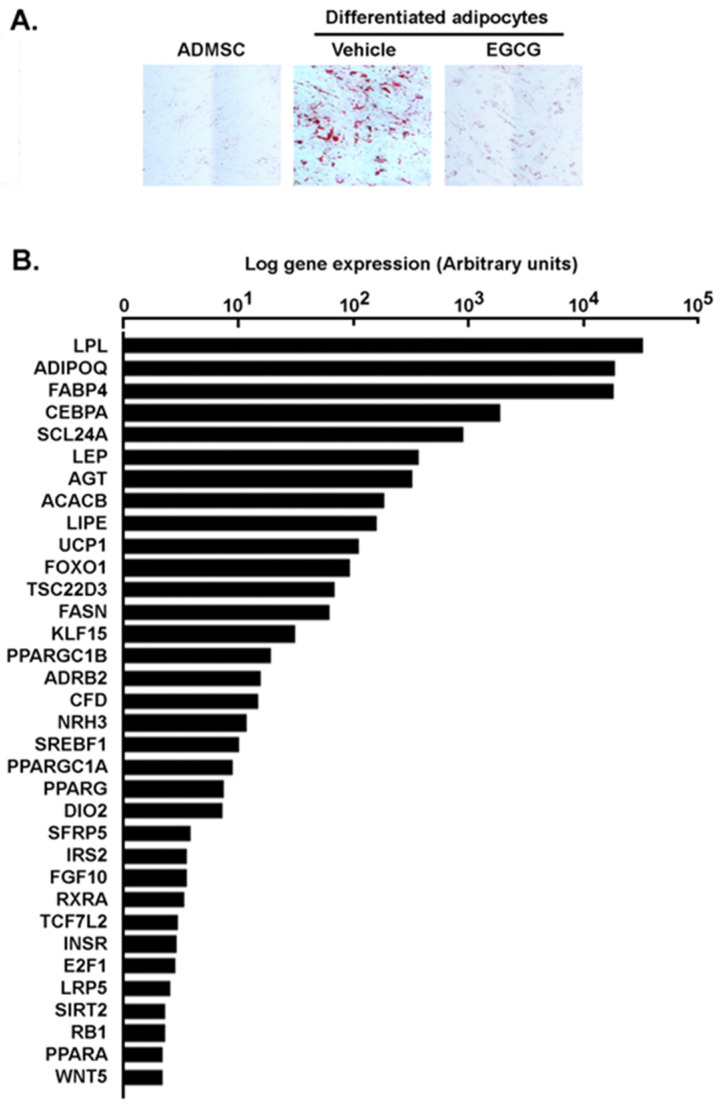

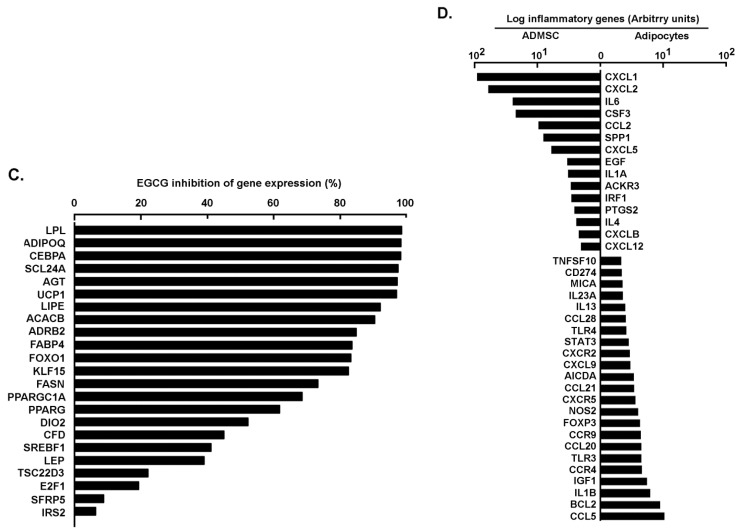
Transcriptional validation of ADMSC differentiation into adipocytes and green tea-derived EGCG inhibition of adipogenesis. (**A**) ADMSC were differentiated into mature adipocytes in the presence or not of 10 μM EGCG as described in the Methods section. Oil Red O staining was performed at different stages of differentiation to confirm mature adipocyte status through lipid droplets formation, typically observed between day 6 and day 12. (**B**) Total RNA was extracted from ADMSC, adipocytes, as well as ADMSC differentiated in the presence or not of EGCG. RT-qPCR was performed using a RT^2^-Profiler gene array to assess adipogenesis gene expression levels. Ratios of adipocytes gene expression on ADMSC gene expression were performed and expressed in a logarithmic scale. (**C**) Ratios of adipocytes differentiated in the presence of EGCG on ADMSC were performed, and EGCG inhibitory potential calculated. (**D**) Total RNA was extracted from ADMSC and from adipocytes. RT-qPCR was performed using a RT^2^-Profiler gene array to assess inflammatory-associated gene expression levels. Ratios of adipocytes/ADMSC gene expression (right), and ADMSC/adipocytes (left) gene expression were performed and expressed in a logarithmic scale. Gene arrays data reflect one representative experiment out of two.

The differentiation of human adipose-derived mesenchymal stem cells (ADMSC) into mature adipocytes was first performed according to the manufacturer’s instructions. Lipid droplet generation was effectively observed as early as day 6 in mature adipocytes as confirmed by increases in size shape due to lipid vesicles accumulation and by Oil Red O staining (Figure 1A). Interestingly, such differentiation processes were abrogated by the presence of the diet-derived epigallocatechin-3-gallate (EGCG), a flavonoid believed to prevent the obesity-associated mortality [19,20], as both cell size and Oil Red O staining levels were reduced (Figure 1A). When total RNA was extracted from each condition and RT-qPCR was performed to assess gene expression levels, classical adipogenesis-associated genes were induced, including the transcription factors PPARγ and C/EBPα, which promote expression of genes that confer the characteristics of mature adipocytes [21]. Among those genes are insulin receptors, fatty acid synthase, lipoprotein lipase, adiponectin, leptin, acetyl-CoA carboxylase beta, and fatty acid binding protein 4 (FABP4, Figure 1B) [22], the latter being a new player connecting obesity with breast cancer development [23]. Interestingly, EGCG inhibited the induction of these genes by 40–80%, suggesting that the resulting mature adipocytes may additionally exhibit an altered secretome profile (Figure 1C). Finally, differential gene expression of inflammatory biomarkers was assessed comparing genes preferentially expressed in ADMSC (Figure 1D, left) versus genes preferentially expressed in adipocytes (Figure 1D, right). Whereas this does not preclude their overall expression, data confirm that the composition of the respective pro-inflammatory secretome characterizes each of the undifferentiated vs. differentiated adipocyte states.

### 2.2. Adipocytes Secretome, but Not that of Adipose-Derived Mesenchymal Stem Cells, Triggers Increased TNBC-Derived Cell Migration

**Figure 2:**
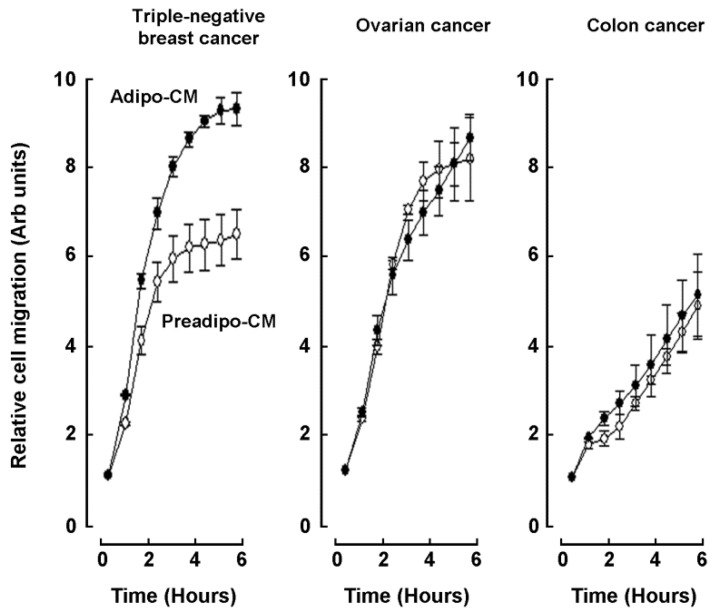
Adipocytes secretome, but not that of ADMSC, triggers increased TNBC-derived cell migration. Real-time cell migration was performed using the xCELLigence System-RTCA as described in the Methods section in response to ADMSC conditioned media (Preadipo-CM, open circles) or adipocytes conditioned media (Adipo-CM, closed circles). Cells assessed were human TNBC-derived MDA-MB-231, ES-2 ovarian carcinoma, and HT-29 colorectal adenocarcinoma.

**Figure 3:**
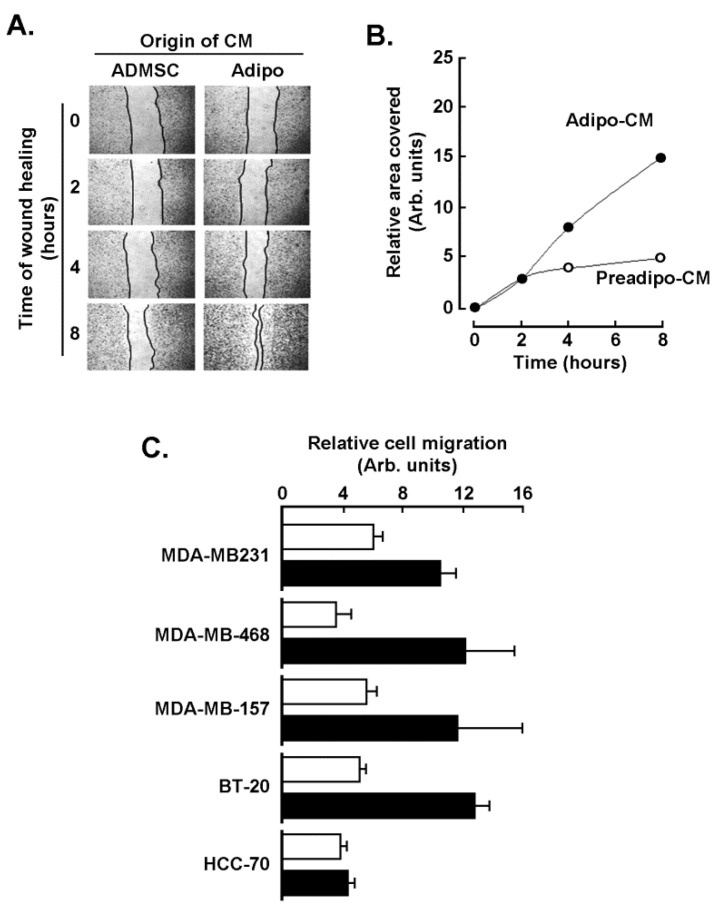
Chemotaxis response of different TNBC-derived cell lines to adipocytes secretome. (**A**) A wound-healing assay was performed with MDA-MB-231 cells in response to conditioned media isolated from ADMSC (Preadipo-CM) and from adipocyte conditioned media (Adipo-CM), and pictures were taken of the recovered area as described in the Methods section. (**B**) The extent of the recovered area was calculated from a representative experiment. (**C**) Chemotaxis screening of four other human TNBC-derived cell lines was performed with MDA-MB-468, MDA-MB-157, BT-20, and HCC-70 cells in response to either Preadipo-CM (white bars) or Adipo-CM (black bars). Two independent cell migration assays were performed and measured in triplicate.

Secretome from ADMSC and from adipocytes cell cultures was defined as the conditioned media (CM) collected from each of the respective cell lines, and chemoattractant capacity was assessed on human TNBC-derived MDA-MB-231 cells, ES-2 ovarian cancer cells, and HT-29 colorectal cancer cells. Adipocytes secretome (Adipo-CM; Figure 2 closed circles) was found to trigger a higher chemotactic response in TNBC cells than in any of the two other cancer cell line models tested when compared to ADMSC secretome (Preadipo-CM; Figure 2, open circles). To further document the evidence of increased chemotaxis observed in MDA-MB-231 cells, a wound-healing assay was performed (Figure 3A), and was found to corroborate the increased invasiveness phenotype as cells recovered the wounded area faster in response to Adipo-CM (Figure 3B). Finally, chemotaxis screening of four other human TNBC-derived cell lines was performed with MDA-MB-468, MDA-MB-157, BT-20, and HCC-70 cells in response to either Preadipo-CM or Adipo-CM. All but HCC-70 cells were found to be more responsive to Adipo-CM than to Preadipo-CM (Figure 3C). This suggests that the mature adipocytes secretome exerts oncogenic paracrine regulation of TNBC cells, increasing their invasiveness.

### 2.3. EGCG Inhibits Both the Acute Response and the Paracrine Regulation of TNBC Cell Chemotaxis Response to Mature Adipocytes Secretome

**Figure 4:**
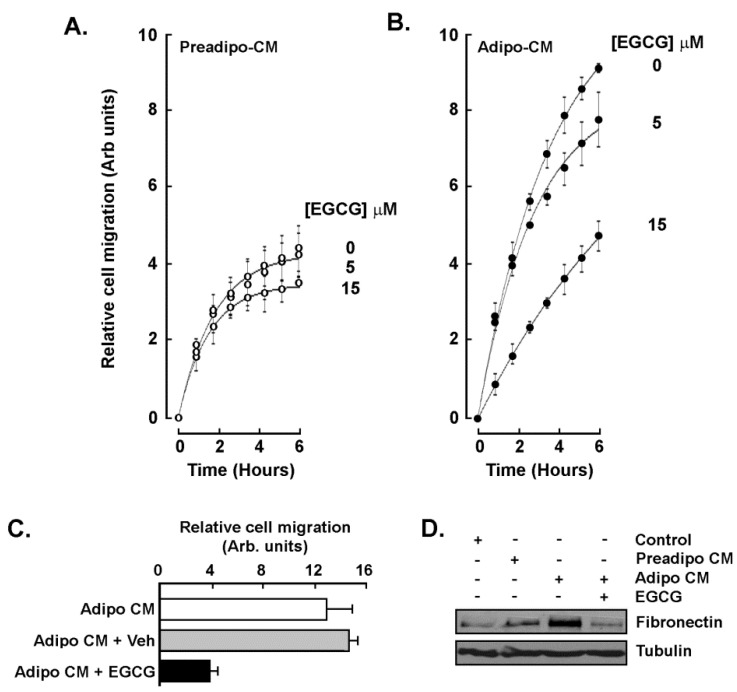
EGCG inhibits the acute response and prevents the paracrine regulation of TNBC cell chemotaxis to adipocytes secretome. MDA-MB-231 cell migration was assessed as described in the Methods section in the presence of increasing concentrations of EGCG and in response to (**A**) conditioned media isolated from ADMSC (Preadipo-CM), or (**B**) conditioned media isolated from differentiated adipocytes (Adipo-CM). (**C**) Chemotaxis response to conditioned media harvested from adipocytes that were differentiated from ADMSC in untreated (white bar), vehicle-treated (ethanol, grey bars), or differentiated in the presence of 15 μM EGCG (black bars). (**D**) MDA-MB-231 cells were treated for 24 h in basal media (Control), Preadipo-CM, Adipo-CM, or Adipo-CM in the presence of 30 μM EGCG. Fibronectin and Tubulin protein expression were then assessed by immunoblotting using the respective cell lysates. Two independent cell migration assays were performed and measured in triplicate.

The capacity of EGCG to regulate the chemotaxis response triggered by the adipocytes secretome was next assessed. Two conditions were tested: first adding the catechin molecule into the Adipo-CM (acute treatment) and second using a CM from ADMSC differentiated in the presence of 10 μM EGCG (paracrine regulation). Whereas little inhibition was found when MDA-MB-231 cells were exposed to Preadipo-CM in the presence of increasing EGCG concentrations (Figure 4A), a dose-dependent inhibition of chemotaxis was observed in response to Adipo-CM and, when treated at 15 μM EGCG, reached migration levels equivalent to the basal Preadipo-CM response (Figure 4B). When MDA-MB-231 chemotaxis response was assessed with CM isolated from mature adipocytes and from ADMSC differentiated in the presence of EGCG, it was also found to be reduced (Figure 4C). In light of the previously reported involvement of the Fibronectin/STAT3 signaling axis in epithelial-mesenchymal transition (EMT), a process that increases invasion and metastasis of breast cancer cells [24], cell lysates of the above conditions were isolated. Immunoblotting was performed and Adipo-CM was found to trigger Fibronectin expression, a condition which was abrogated by EGCG (Figure 4D). Altogether, these data suggest that specific signaling pathway inhibition may account for the acute EGCG inhibition of chemotaxis. More importantly, EGCG is able to prevent the paracrine regulation of TNBC cell chemotaxis response by altering the adipocyte secretome profile and, ultimately, the acquisition of an EMT-related invasive phenotype.

### 2.4. STAT3 Is Involved in the Paracrine Chemotaxis Response to Adipocytes Secretome

**Figure 5:**
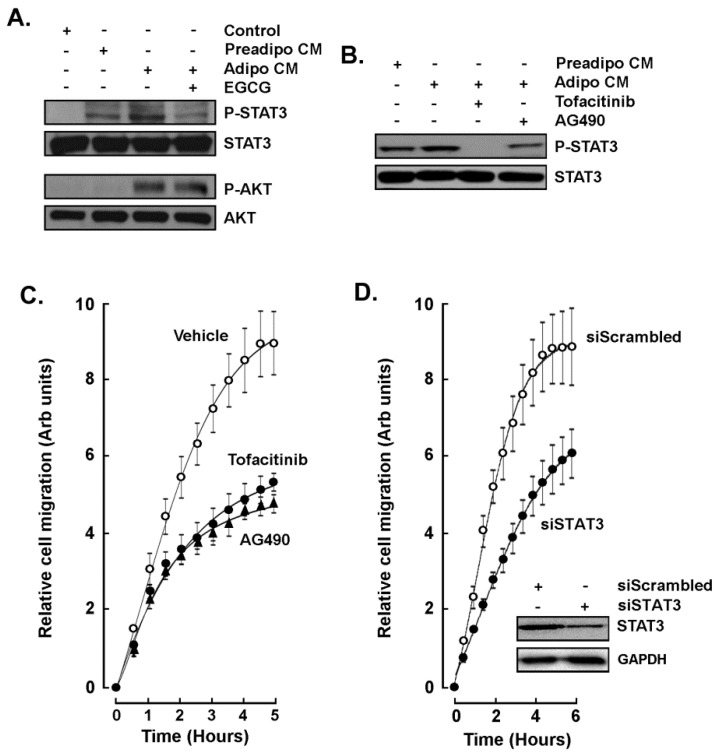
STAT3 involvement in the paracrine chemotaxis response to adipocytes secretome. (**A**) MDA-MB-231 cells were treated for 24 h in the presence of basal media (Control), ADMSC conditioned media (Preadipo-CM), adipocyte conditioned media (Adipo-CM), or Adipo-CM in the presence of 30 μM EGCG. STAT3 and AKT phosphorylation status was then assessed by immunoblotting using the respective cell lysates. (**B**) Cells were similarly treated as in (**A**) with the difference that 30 μM Tofacitinib or AG490 were added to the CM. (**C**) MDA-MB-231 chemotaxis response to conditioned media harvested from adipocytes was performed as described in the Methods section in the absence (vehicle), or presence of Tofacitinib (closed circles), or AG490 (closed triangles). (**D**) MDA-MB-231 chemotaxis response to conditioned media harvested from adipocytes was performed as described in the Methods section in control siScrambled cells (open circles), or in siSTAT3-transfected cells (closed circles). Insert shows a representative Western blot monitoring the extent of STAT3 silencing from siScrambeled and siSTAT3-transfected cells. GAPDH was used as a loading control.

Stress-induced signaling through signal transducer and activator of transcription 3 (STAT3) is, among other transducing pathways, involved in the initiation, progression, metastasis, and immune evasion of TNBC [25]. More recently, STAT3 is further considered as a potential therapeutic target [26,27,28]. Whether STAT3 is involved in the paracrine response to mature adipocytes secretome was next assessed. MDA-MB-231 cells were exposed to Preadipo-CM or to Adipo-CM for 24 h and STAT3 phosphorylation status assessed by Western blotting. STAT3 phosphorylation was induced in response to Preadipo-CM, whereas phosphorylation was higher in response to Adipo-CM (Figure 5A, upper panels). Interestingly, phosphorylation of the serine/threonine-specific protein kinase AKT, which plays a key role in multiple cellular processes such as cell proliferation, transcription, and cell migration [29,30], was only induced in response to Adipo-CM (Figure 5A, lower panels). However, EGCG was able to inhibit STAT3 but not AKT phosphorylation. In addition to EGCG, Tofacitinib and AG490, two pharmacological inhibitors of STAT3 phosphorylation [31,32], also inhibited Adipo-CM-induced STAT3 phosphorylation (Figure 5B) as well as the MDA-MB-231 chemotactic cell response (Figure 5C). Finally, transient siRNA-mediated gene silencing of STAT3 was performed in MDA-MB-231 cells in order to reduce the STAT3 protein content (Figure 5D, insert). Then, siScrambled or siSTAT3 cells were challenged to migrate in response to Adipo-CM as a chemoattractant. Similarly to STAT3 pharmacological inhibition, transient silencing of STAT3 was found to reduce the MDA-MB-231 chemotaxis response to the adipocytes secretome (Figure 5D).

### 2.5. Adipocytes Secretome Triggers In Vitro 3D-Capillary-Like Structure Maturation, and STAT3 Inhibition Prevents such Maturation

**Figure 6:**
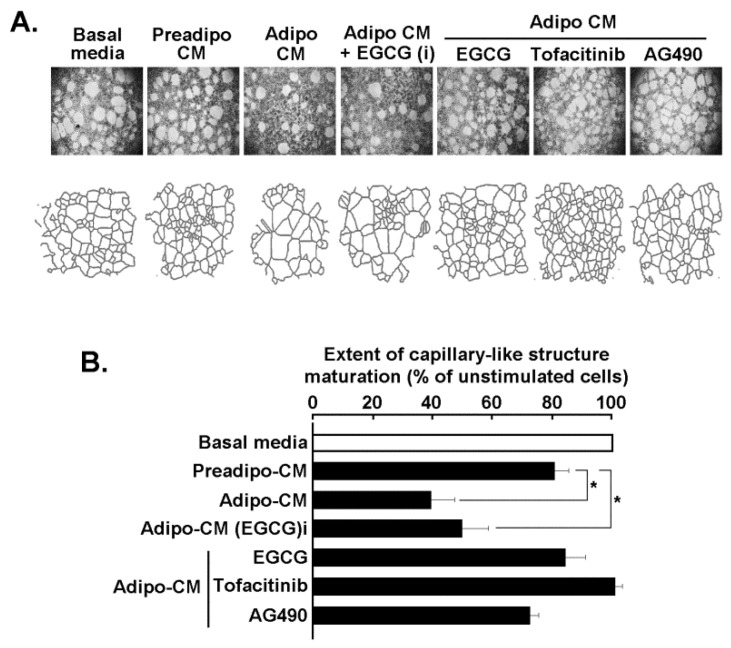
Adipocytes secretome triggers in vitro 3D-capillary-like structure maturation, and STAT3 inhibition prevents such maturation. (**A**) MDA-MB-231 TNBC-derived cells were seeded on Matrigel and cultured for 24 h as described in the Methods section. Representative pictures were taken (upper panels), and digitalized structures used for the analysis presented (lower panels). (**B**) Analysis of the 3D capillary-like structure maturation on Matrigel was performed using ImageJ, and was assessed in the presence of either serum-deprived basal media (unstimulated condition), or stimulation with Preadipo-CM, Adipo-CM, or Adipo-CM (i) (CM harvested upon ADMSC differentiation in the presence of 30 μM EGCG). Acute additions of EGCG, Tofacitinib, or AG490 (all at 30 μM) were done in the presence of Adipo-CM to the cell culture. Significance: * *p* < 0.05.

The paracrine regulation of ADMSC and of mature adipocytes secretome was next assessed on in vitro vasculogenic mimicry (VM), a process known to in part be responsible for TNBC chemotherapy resistance [33]. MDA-MB-231 cells were seeded on Matrigel and cultured for 24 h as described in the Methods section in order to generate 3D capillary-like structures. Representative pictures were taken (Figure 6A, upper panels), and digitalized structures were used for the analysis presented (Figure 6A, lower panels). Analysis of the 3D capillary-like structure maturation on Matrigel was performed (Figure 6B) and shows that unstimulated cells cultured on Matrigel and in the presence of serum-deprived basal media (unstimulated condition) led to the formation of 3D structures, which was accelerated by the Preadipo-CM and furthermore by the Adipo-CM (stimulated conditions). The Adipo-CM generated with EGCG added during adipocyte differentiation (Adipo-CM + EGCG (i)), was ineffective in preventing 3D structure maturation. On the contrary, acute additions of EGCG, Tofacitinib, or AG490, were all shown to prevent STAT3 phosphorylation in response to adipocyte secretome, and effectively altered VM processes. Taken together, these results suggest that efficient targeting of the STAT3 signaling pathway may be achieved through diet-derived polyphenols and can prevent the acquisition of an aggressive phenotype in MDA-MB-231 cells, a model of the TNBC.

## 3. Discussion

Adipogenesis is a critical step in adipocyte physiology, and consists in the terminal differentiation of adipocyte precursor cells (pre-adipocytes) into adipocytes that allows increased storage of fatty acids [34]. Here, ADMSC differentiation into mature adipocytes has been validated by increased expression of PPARγ and C/EBPα, two transcription factors considered among the master regulators of this process [35]. Interestingly, expression of both biomarkers was prevented by EGCG (Figure 1), and consequently, it was anticipated that this would alter the state of differentiation as well as the secretome profile of mature adipocytes. Accordingly, distinct pro-inflammatory profiles are shown to characterize the ADMSC and adipocyte respective phenotypes (Figure 1D). In accordance with previous studies, IL6 was more expressed in ADMSC than in mature adipocytes [36], and the expression of NOS2, IGF1, and IL1B were higher in mature adipocytes than in ADMSC [37,38,39]. Apart from the regulation of the body’s energy balance, factors secreted from adipose tissue in obesity play key roles in the modulation of metabolic processes, insulin sensitivity and immunological responses [40], and are believed to provide protumorigenic chemokines to promote breast cancer progression [41]. Unfortunately, the detailed mechanisms involved in adipose tissue paracrine regulation of breast cancer cells are still not well understood.

Here, we provide evidence for a specific and increased paracrine regulatory impact of the adipocytes secretome on several TNBC-derived cell models. Chemotaxis response was found to be significantly induced by the secretome of differentiated adipocytes when compared to that of undifferentiated adipocytes, and this required the activation of the AKT and STAT3 signaling pathways. EGCG, as well as JAK/STAT inhibitors Tofacitinib and AG490, all prevented the increase in chemotactic response to cytokines and growth factors originating from mature adipocytes. Intriguingly, AKT phosphorylation was also induced but could not be prevented by EGCG. Whereas AKT-targeted therapy is believed to be a promising strategy to overcome drug resistance in breast cancer [30], such selective targeting of signaling pathways by EGCG prompts for more research.

The adipose microenvironment in obese people bears many similarities with the tumor microenvironment with respect to associated cellular composition, chronic low-grade inflammation, and a high ratio of reactive oxygen species to antioxidants [9]. In addition, the secretion of a number of inflammation-related adipokines is upregulated by hypoxia, which is present in some areas of the expanded adipose tissue [42]. Hence, obesity creates a pro-inflammatory environment that is believed to favor the incidence of several cancers [43] through numerous signal transduction pathways, including the JAK/STAT3 pathway [44]. Targeting oncogenic transcription factors by polyphenols has recently been inferred [45], and inhibition of JAK/STAT3 transducing events by EGCG has been reported in numerous cancers [46,47,48]. More recently, emerging evidence of dietary phytochemicals in our fight against cancer has ascribed a role in targeting cancer stem cells (CSC) often associated with chemoresistance and cancer recurrence [49,50]. Such an avenue towards CSC targeting has prompted preclinical and clinical settings to repurpose pre-existing drugs to treat TNBC on the basis of molecular mechanisms and signaling pathways such as STAT3 [51,52]. In our study, we demonstrate that EGCG prevents the differentiation of ADMSC and modulates the secretome profile of these cells. Furthermore, once the cells are fully mature, EGCG can hinder its paracrine regulation over the TNBC cells within the tumor microenvironment, which highlights the potential benefit of its consumption.

Paracrine-mediated regulation of an increased invasive phenotype can, in part, involve EMT processes [53]. Here, we show that part of the chemotactic response could be achieved through such process as the expression of the EMT biomarker Fibronectin and was induced upon incubation with differentiated adipocytes secretome, but not with that from the ADMSC. Interestingly, EGCG prevented both the chemotactic response of TNBC cells as well as the induction of Fibronectin in accordance with its capacity to inhibit EMT [54]. EGCG targeting of EMT processes has also recently been documented in ES-2 ovarian cancer cells where cell migration and in vitro VM were abrogated [55]. In the current study, we demonstrate that the paracrine regulation of MDA-MB-231-mediated in vitro VM was abrogated by all three JAK/STAT3 inhibitors (EGCG, Tofacitinib, AG490) tested.

Adipocytes, and their precursors ADMSC, are thought to sustain tumor phenotypes in part through secretion of signaling molecules and vesicles containing proteins, lipids and nucleic acids [56]. On the other hand, during their interaction with cancer cells, ADMSC can in return be reprogrammed into cancer-associated adipocytes to further secrete adipokines, which stimulate the adhesion, migration, and invasion of cancer cells [57]. Such bidirectional communication between adipose and breast cancer cells has laid foundations for the recruitment of macrophages to the mammary tumor inflammatory microenvironment through increased release of cytokines, growth factors and extracellular matrix components [58,59]. Our findings are in accordance with the previous statements, as we found that both ADMSC and mature adipocytes secrete factors that could contribute to tumor development like IL6, EGF, PTGS2 and IL4 in ADMSC, and CCL5, IL1β and IGF in adipocytes. Recently, FABP4, a member of a family of circulating adipose fatty acid binding proteins, has emerged as a new link in the obesity-associated breast/mammary tumor development [23,60]. Of particular interest, circulating blood concentration FABP4 levels have been proposed as a new independent breast cancer biomarker as it was found increased in breast cancer patients [61]. Here, we report that FABP4 transcript levels were increased upon adipocyte differentiation and, more importantly, that such an induction can efficiently be prevented by EGCG. More studies will be needed to identify, among the secreted factors, which one(s) have a leading role in order to design pharmacological interventions.

In conclusion, this study first validated our preadipocytes differentiation protocol at both the cellular and transcriptional levels. Both the cellular staining and the transcriptomic data demonstrated clear modulation by EGCG. This last evidence strongly suggests that, although beyond the immediate scope of this study, the overall soluble secreted growth factors (secretome) from preadipocytes and mature adipocytes may trigger differential chemotactic response. The exact identification of these individual growth factors/cytokines would be a logical follow-up although the current approach used, which is the use of the cells’ conditioned media to reflect the synergistic action of the complete actors of the secretome, better reflects the pathophysiological tissue microenvironment. One may envision later and address of the impact of hypoxia, and EGCG tissue biodistribution/bioavailability using in vivo approaches, as these remain major concerns in obesity.

**Figure 7:**
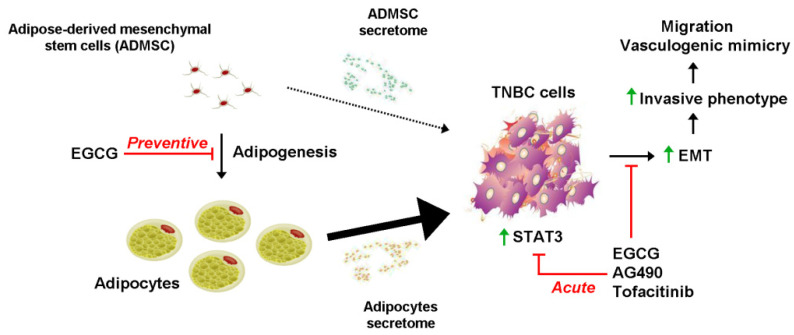
Scheme summarizing the preventive and acute effects of EGCG on STAT3-mediated acquisition of an invasive TNBC phenotype. Adipogenesis is induced during ADMSC differentiation into mature adipocytes. Both of the cell types have characteristic and distinct secretome profiles composed of different levels of pro-inflammatory cytokines, chemokines, and growth factors (Figure 1D). Whereas ADMSC secretome was characterized by some chemotactic properties towards TNBC cells (dotted arrow), this was significantly triggered by the secretome resulting from mature adipocytes (large arrow). EGCG was able to prevent such an effect by inhibiting adipogenesis (Preventive experimental condition). Increased EMT explains, in part, the resulting increases in TNBC cell migration and vasculogenic mimicry in response to the adipocytes secretome, the response of which can be reduced through the inhibition of STAT3-mediated signaling by EGCG, AG490, and Tofacitinib (Acute experimental condition).

Our study further provides new molecular evidence demonstrating how dietary intervention upon adipogenesis could alter the secretome profile during adipocyte maturation, and the paracrine regulation of TNBC cell acquisition of an invasive phenotype (Figure 7). Data also highlight a critical role of the JAK/STAT3 signaling pathway in cell chemotaxis and VM, which can be targeted by EGCG as efficiently as by the pharmacological agents Tofacitinib and AG490. Preventing the onset of an obesogenic environment should help reduce the incidence of TNBC development.

## 4. Materials and Methods

### 4.1. Materials

Sodium dodecylsulfate (SDS), epigallocatechin-3-gallate (EGCG), Tofacitinib, AG490, and bovine serum albumin (BSA) were purchased from Sigma-Aldrich Canada (Oakville, ON). Electrophoresis reagents were purchased from Bio-Rad (Mississauga, ON, Canada). The enhanced chemiluminescence (ECL) reagents were from Amersham Pharmacia Biotech (Baie d’Urfé, QC, Canada). Micro bicinchoninic acid protein assay reagents were purchased from Pierce (Rockford, IL, USA). The polyclonal antibodies against Fibronectin, Tubulin, AKT, phosphorylated AKT, STAT3 and phosphorylated STAT3 were obtained from Cell Signaling Technology Inc. (Danvers, MA, USA). Horseradish peroxidase-conjugated donkey anti-rabbit and anti-mouse IgG secondary antibodies were obtained from Jackson ImmunoResearch Laboratories (West Grove, PA, USA).

### 4.2. Cell Culture

Human serous carcinoma-derived ES-2 ovarian cancer cells, HT-29 colon adenocarcinoma, and triple-negative breast cancer cell lines, including MDA-MB-231, MDA-MB-468, MDA-MB-157, BT-20 and HCC-70, were all purchased from the American Type Culture Collection (ATCC). BT-20, ES-2 and HT-29 cells were grown as monolayers in McCoy’s 5a Modified Medium (Wisent, 317-010-CL) containing 10% fetal bovine serum (Life Technologies, 12483-020), 100 U/mL penicillin and 100 mg/mL streptomycin (Wisent, 450-202-EL). MDA-MB-231, MDA-MB-157 and MDA-MB-468 breast cancer cell lines were grown in DMEM Medium (Wisent, 319-005-CL) supplemented with 10% of fetal bovine serum, while HCC-70 cells were cultured in RPMI (Wisent, 350-007-CL). All cells were cultured at 37 °C under a humidified 95%–5% (*v*/*v*) mixture of air and CO_2_.

### 4.3. Adipose-Derived Mesenchymal Stem Cell Differentiation

Adipose-derived mesenchymal stem cells (ADMSC; ATCC, PCS-500-011) were grown using the Mesenchymal Stem Cell Basal Medium (ATCC, PCS-500-030), and supplemented with Mesenchymal Stem Cell Growth Kit for adipocyte differentiation- Low Serum (ATCC, PCS-500-040). When cells reached 70–80% confluency, they were seeded at a density of 18,000 cells/cm^2^ and differentiated into mature adipocytes with Adipocyte Differentiation Toolkit (ATCC, PCS-500-050) following the manufacturer’s instructions. Briefly, cells were incubated at 37 °C with 5% CO_2_ for 48 h before initiating adipocyte differentiation. Then, media was removed and cell monolayers rinsed with room temperature DPBS (ATCC, 302200). Next, 2 mL (for 6-well plates) of pre-warmed (37 °C) adipocyte differentiation/initiation medium was added to each well to initiate adipocyte differentiation. After a 48 h incubation, half the volume of media was removed and the previous steps repeated for another 48 h. Later, the maintenance phase was initiated by carefully removing 2 mL of media from each well (leaving 1 mL), and replacing it with 2 mL of pre-warmed adipocyte differentiation/maintenance medium in each well. The latter step was repeated every 3–4 days for another 11 days until adipocytes reached full maturity (~12–15 days). Conditioned media (CM) was collected at different time points.

### 4.4. Oil Red O Staining

In order to assess adipocyte maturation status and to visualize the lipid droplet formation, medium was removed and cells were incubated with 10% formalin at room temperature for 5 min, then fresh formalin was added and cells were store at 4 °C in the dark, up to 2 days. The wells were next washed with 60% isopropanol and left to dry. Oil Red O (0.5 g/100 isopropanol stock solution; Sigma-Aldrich) was added and left for 10 min. Wells were finally washed with water four times and pictures taken.

### 4.5. Total RNA Isolation, cDNA Synthesis and Real-Time Quantitative PCR

Total RNA was extracted from cell monolayers using TriZol reagent (Life Technologies, Gaithersburg, MD, USA). For cDNA synthesis, 2 μg of total RNA were reverse-transcribed using a high capacity cDNA reverse transcription kit (Applied Biosystems, Foster City, CA, USA). cDNA was stored at −80 °C prior to PCR. Gene expression was quantified by real-time quantitative PCR using iQ SYBR Green Supermix (Bio-Rad, Hercules, CA, USA). DNA amplification was carried out using an Icycler iQ5 (Bio-Rad, Hercules, CA, USA) and product detection was performed by measuring binding of the fluorescent dye SYBR Green I to double-stranded DNA.

### 4.6. Human Adipogenesis and Inflammation PCR Arrays

The Human Adipogenesis RT^2^ Profiler PCR Array (PAHS-049Z) and the Human Cancer Inflammation & Immunity Crosstalk RT^2^ Profiler™ PCR Array (PAHS-181Z) were used according to the manufacturer’s protocol (QIAGEN). The detailed list of the key genes assessed can be found on the manufacturer’s website (https://geneglobe.qiagen.com/us/product-groups/rt2-profiler-pcr-arrays; accessed on 13 February 2021). Using real-time quantitative PCR, we reliably analyzed expression of a focused panel of genes related to adipogenesis and PPARγ targets, or to inflammatory cytokines/receptors. Relative gene expressions were calculated using the 2^− ΔΔC^_T_ method, in which C_T_ indicates the fractional cycle number where the fluorescent signal reaches detection threshold. The “delta–delta” method uses the normalized ΔC_T_ value of each sample, calculated using a total of five endogenous control genes (*B2M*, *HPRT1*, *RPL13A*, *GAPDH,* and *ACTB*). Fold change values are then presented as average fold change = 2(average ^ΔΔC^_T_) for genes in differentiated adipocytes relative to pre-adipocytes. Detectable PCR products were obtained and defined as requiring <35 cycles. The resulting raw data were then analyzed using the PCR Array Data Analysis Template (http://www.sabiosciences.com/pcrarraydataanalysis.php; accessed on 1 February 2021). This integrated web-based software package automatically performs all ΔΔC_T_-based fold-change calculations from the uploaded raw threshold cycle data.

### 4.7. Western Blotting

TNBC-derived MDA-MB-231 cells were lysed in a buffer containing 1 mM each of NaF and Na_3_VO_4_, and proteins (30 μg) were separated by SDS-polyacrylamide gel electrophoresis (PAGE). After electrophoresis, proteins were electro-transferred to polyvinylidene difluoride membranes, which were then blocked for 1 h at room temperature with 5% nonfat dry milk in Tris-buffered saline (150 mM NaCl, 20 mM Tris-HCl, pH 7.5) containing 0.3% Tween-20 (TBST; Bioshop, TWN510-500). Membranes were further washed in TBST and incubated with the appropriate primary antibodies (1/1000 dilution) in TBST containing 3% BSA and 0.1% sodium azide (Sigma-Aldrich), followed by a 1 h incubation with horseradish peroxidase-conjugated donkey anti-rabbit or anti-mouse IgG at 1/2500 dilutions in TBST containing 5% nonfat dry milk. Immunoreactive material was visualized by ECL.

### 4.8. Chemotactic Cell Migration Assay

Cell migration assays were carried out using the Real-Time Cell Analyzer (RTCA) Dual-Plate (DP) Instrument of the xCELLigence system (Roche Diagnostics). Adherent cell monolayers were trypsinized and seeded (30,000 cells/well) onto CIM-Plates 16 (Roche Diagnostics). These migration plates are similar to conventional Transwells (8 μm pore size) but have gold electrode arrays on the bottom side of the membrane to provide real-time measurement of cell migration. Prior to cell seeding, the underside of the wells from the upper chamber were coated with 25 μL of 0.15% gelatin in PBS and incubated for 1 h at 37 °C. Chemotaxis was monitored for 8 h using as chemoattractant either media conditioned from serum-starved ADMSC (Preadipo-CM) or from mature adipocytes (Adipo-CM), in the presence or not of EGCG, Tofacitinib, or AG490. The impedance values were measured by the RTCA DP Instrument software and were expressed in arbitrary units as Normalized Cell Migration Index. Each experiment was performed three times in duplicate.

### 4.9. Wound-Healing Assay

MDA-MB-231 cells were seeded into 6-well tissue culture dishes and grown to nearly confluent cell monolayers. Then, a linear wound was generated in the monolayer with a sterile 200 μL pipette tip creating a cell-free area [62]. Cultures were gently washed with the growth medium to remove loose cells. The cells were then treated with either Preadipo-CM or Adipo-CM. Immediately after the scratch and at 2, 4, and 8 h, at least four images of the scraped area were captured using phase contrast microscopy and analyzed using NIH ImageJ software [63]. Two independent experiments were performed, using three wells for each stimulating condition. The same scratched area was selected for the measurements at each time of the study.

### 4.10. In Vitro Vasculogenic Mimicry Assay

In vitro vasculogenic mimicry (VM) of human TNBC-derived MDA-MB-231 cells was assessed by Matrigel tube formation as previously described [55]. In brief, each well of a 96-well plate was pre-coated with 50 μL of Matrigel. After gel solidification, MDA-MB-231 cell suspension in culture media (5 × 10^4^ cells/200 μL) was seeded into the wells. Either serum-deprived basal media (unstimulated condition), or stimulation with Preadipo-CM or Adipo-CM (stimulated conditions) were performed. Acute additions of EGCG, Tofacitinib, or AG490 (all at 30 μM) were done in the presence of Adipo-CM to the cell culture and incubated at 37 °C in a CO_2_ incubator. Pictures were taken overtime using a digital camera attached to a phase-contrast inverted microscope. Images were then placed in bins and subjected to the “Skeletonize” function of ImageJ software. The corresponding loop area, loop perimeter, branching, and tube elongation parameters were measured using the 2D/3D skeleton PlugIn [64] for the NIH ImageJ software [65].

### 4.11. Statistical Data Analysis

Unless otherwise stated, data and error bars were expressed as means ± SEM of three or more independent experiments. Statistical analysis of data was performed by Kruskal–Wallis with DunnTukey’s post-test to establish differences among groups or Mann–Whitney for two groups’ comparison. Probability values of less than 0.05 were considered significant and an asterisk identifies such significance in the figures. GraphPad Prism 7 software (San Diego, CA, USA) was used for all analyses.

## Data Availability

All data generated or analyzed during this study are included in this published article.

## Sample Availability

Samples of the compounds are not available from the authors.

## Abbreviations

ADMSC	Adipose-derived mesenchymal stem cells
AKT	Protein kinase B
C/EBPα	CCAAT/enhancer-binding protein alpha
CM	Conditioned media
CSC	Cancer stem cells
EGCG	Epigallocatechin-3-gallate
EMT	Epithelial-mesenchymal transition
FABP4	Fatty acid binding protein 4
PPARγ	Peroxisome proliferator-activated receptor gamma
STAT3	Signal transducer and activator of transcription 3
TNBC	Triple negative breast cancer
VM	Vasculogenic mimicry
